# News and Coming Events

**Published:** 1908-12-19

**Authors:** 


### News and Coming Events.

The King has been graciously pleased to sanction the promotion in the Grand Priory of the Order of the Hospital of St. John of Jerusalem in England of Major Sir Allan Perry, M.D., D.P.H., as a Knight of Grace.

According to the St. Petersburg correspondent of the *Times*, writing on December 13, the Russian Government has at last decided to take steps to protect St. Petersburg from another cholera epidemic. The municipal sanitary authorities, who have revealed their incapacity in the matter, have been placed under the control of a Government official.

The Nobel Prize Committees have decided to award the prizes this year as follows: Chemistry.—Professor Ernest Rutherford, of Manchester University. Physics.—Professor Gabriel Lippmann, Paris. Medicine.—Prize divided between Professor Metchnikoff, of Paris, and Professor Paul Ehrlich, of Frankfurt-on-Main. Literature.—Professor Rudolf Eucken, of Jena.

The Council of the University of Manchester has appointed Dr. Henry Richmond Hutton, M.A., M.B., lecturer in diseases of children in the University. Dr. Hutton graduated in medicine at Cambridge in 1881, and was for some time demonstrator of physiology and pathology at St. Thomas's Hospital, London. For 26 years he has held the position of physician to the Manchester Children's Hospital.

At the last meeting of the Cardiff Infirmary board of management it was reported that there was an overdraft of £20,000 at the bank; but it was announced at the same time that Lord Aberdare had promised to become responsible for any overdraft up to £25,000. A scheme has been inaugurated by Lady Aberdare to raise a fund of 1,000,000 shillings for the infirmary, which will be known as the Lady Aberdare Million Shillings League.

With the object of creating interest locally in the work of the After-Care Association, which does very useful work on behalf of poor persons discharged recovered from asylums for the insane, a meeting was held in the hall of Wadham College, Oxford, on December 7. In the unavoidable absence of the Bishop, the Warden of Wadham presided. The Secretary (Mr. H. Thornhill Roxby) explained the objects of the society, and addresses were given by Dr. J Neil and Miss Davenport Hill.

The Medical Officer of Health for the City of London in his quarterly report, referring to preservatives in food, says that "The physiological action of boric acid has been investigated by many observers, and the evidence now available enables a juster estimate to be formed with regard to its action on the human system. Upon a review of all facts there can be no doubt that when this or any other drug is added to a food product the purchaser should be notified of its presence. One grave objection to the use of boric acid is that it affords facilities for utilising meat in which decomposition has commenced, as the evidence of putrefaction change is masked by the antiseptic properties of this acid, which has no pronounced smell or taste. It has been found by experiment that, provided the conditions as to locality, general cleanliness, and character of the meat and bread are satisfactory, not less than 26.24 grains of boric acid per pound of sausage material are required to arrest decomposition for three days."

Dr. D. W. Carmalt Jones, M.B. Oxon., M.R.C.P., has been appointed Assistant Physician to Westminster Hospital.

Dr. Comyns Berkeley, M.B., B.C. Cantab., M.R.C.P., has been appointed an Examiner in Midwifery and Diseases of Women to the University of Oxford.

The treasurers of the Middlesex Hospital have received from Mr. Richard R. Hollins a further donation of £100 for the maintenance of the "Hollins Scholarship" attached to the Cancer Research Laboratories.

Under the will of the late Mr. William Knox, of Aberdeen, the University of Aberdeen has received a legacy of £5,000 for the foundation of scholarships in the Faculties of Arts, Divinity, and Medicine, and regulations for the award of these scholarships have now been drawn up. The scholarship in Arts, which will be of the value of over £60 per annum, is to be conferred on the best student who takes the degree of M.A. with double honours in any year.

A very successful Australian Medical Congress has lately been held in Melbourne, every State in the Commonwealth being well represented. A prominent position was given to the discussion of Hospital Management. The subject of "intermediate hospitals" has been largely brought forward by the daily papers, but, according to our Melbourne correspondent, nothing has been suggested that could in any way show how patients are to be well nursed and delicately fed in up-to-date hospitals at less than £2 2s. per week.

At the last annual meeting of the Liverpool University Court a rearrangement of the teaching of the subjects of therapeutics, pharmacology, materia medica, and pharmacy was sanctioned. The subject of therapeutics is now separated from that of pharmacology and a new chair of therapeutics has been established, to which Dr. John Hill Abram has been appointed. Pharmacology will in future be taught as a laboratory course, chiefly by means of demonstrations of the action of drugs. For the purpose of this course and for the conduct of pharmacological researches in the University a special department of pharmacology is in course of establishment. Mr. W. Thelwall Thomas has had conferred upon him the title of associate professor of surgery on the recommendation of the Senate.

At the meeting of the National League for Physical Education and Improvement in the Jerusalem Chamber, Westminster, on December 11, the following resolution was moved and supported by the Archbishop of Canterbury and the Lord Mayor: "That this meeting heartily approves of the action of the League in urging upon the local authorities the appointment of health visitors." The Inter-Departmental Committee on Physical Deterioration pointed out in its report the necessity for co-operation between some central body (such as the League now is) and municipal activity in supervising and directing the work of saving the lives of the children of the nation. Since then much good work has been done by the League, but the efforts have lacked municipal support and the funds which such support brings. The rate of infant mortality remains appalling, and to counteract and remove the ignorance and carelessness of the mothers would be among the duties of the health visitors whom the League is working to see appointed.

